# Remedies

**Published:** 1884

**Authors:** 


### REMEDIES.

Abs. Absinthium.
Acal. Acalypha Indica.
Acet. ac. Aceticum acidum.
Acon. l. Aconitum lycoctonum.
Acon. Aconitum napellus.
Æsc. h. Æsculus hippocastanum.
Agar. Agaricus muscarius.
Agn. Agnus castus.
Alco. Alcohol.
All. c. Allium cepa.
All. s. Allium sativum.
Aloe. Aloe socotrina.
Alumn. Alumen.
Alum. Alumina.
Ambr. Ambra grisea.
Ammc. Ammoniacum.
Am. bro. Ammonium bromatum.
Amm. c. Ammonium carbonicum.
Amm. cau. Ammonium causticum.
Amm. m. Ammonium muriaticum.
Aml. n. Amyl nitrite.
Anac. Anacardium.
Ang. Angustura.
Ant. cr. Antimonium crudum.
Ant. ox. Antimonium oxidum.
Ant. t. Antimonium tartaricum.
Aphis. Aphis chenopodii glauci.
Apis. Apis mellifica.
Apoc. c. Apocynum cannabinum.
Aqu. petr. Aqua petra.
Aral. Aralia racemosa.
Aran. d. Aranea diadema.
Arg. Argentum metallicum.
Arg. c. Argentum cyanidum.
Arg. n. Argentum nitricum.
Arn. Arnica.
Ars. Arsenicum album.
Ars. iod. Arsenicum iodatum.
Arum i. Arum italicum.
Arum m. Arum maculatum.
Arum tr. Arum triphyllum.
Arun. Arundo mauritanica.
Asaf. Asafœtida.
Asar. Asarum.
Asc. t. Asclepias tuberosa.
Asim. Asimina triloba.
Asp. }
Aspar. } Asparagus.
Atro. Atropinum.
Aur. Aurum.
Aur. m. Aurum muriaticum.
Aur. m. n. Aurum muriaticum natronatum.
Bad. Badiaga.
Bals. Balsamum Peruvianum.
Bapt. Baptisia.
Bart. Bartfelder.
Bar. Baryta carbonica.
Bar. m. Baryta muriatica.
Bell. Belladonna.
Benz. Benzinum.
Benz. ac. Benzoicum acidum.
Berb. Berberis.
Bism. Bismuthum.
Bon. Bondonneau.
Bor. Borax.
Both. Bothrops lanceolatus.
Bov. Bovista.
Brom. Bromium.
Bry. Bryonia.
Buf. Bufo.
Cact. Cactus grandiflorus.
Cad. Cadmium sulphuricum.
Cai. Cainca.

Caj. Cajuputum. (Oleum Cajuputi.)
Calad. Caladium.
Calc. ac. Calcarea acetica.
Calc. Calcarea carbonica.
Calc. cau. Calcarea caustica.
Calc. ph. Calcarea phosphorica.
Calc. s. Calcarea sulphurica.
Calo. Calotropis.
Camph. Camphora.
Cann. i. Cannabis indica.
Cann. s. Cannabis sativa.
Canth. Cantharis.
Caps. Capsicum.
Carbo an. Carbo animalis.
Carbo v. Carbo vegetabilis.
Carb. ac. Carbolicum acidum.
Carbn. cl. Carboneum chloratum.
Carbn. h. Carboneum hydrogenisatum.
Carbn. ox. Carboneum oxygenisatum.
Carbn. s. Carboneum sulphuratum.
Card. b. Carduus benedictus.
Carl. Carlsbad.
Casc. Cascarilla.
Castor. Castoreum.
Caul. Caulophyllum.
Caust. Causticum.
Ced. Cedron.
Cent. Centaurea tagana.
Cepa. Allium cepa.
Cham. Chamomilla.
Chel. Chelidonium majus.
Chenop. See Aphis etc.
Chim. Chimaphila.
Chin. China.
Chin. s. Chininum sulphuricum.
Chlo. Chlorum.
Chro. ac. Chromium acidum.
Cic. v. Cicuta virosa.
Cimex. Cimex lectularius.
Cimic. Cimicifuga.
Cina. Cina.
Cinch. Cinchoninum sulphuricum.
Cinnab. Cinnabaris.
Cis. Cistus.
Cle. Clematis erecta.
Coca. Coca.
Cocc. Cocculus.
Coc. c. Coccus cacti.
Cod. Codeinum.
Coff. Coffea cruda.
Colch. Colchicum.
Coll. Collinsonia.
Coloc. Colocynthis.
Colocn. Colocynthinum.
Com. Comocladia dentata.
Con. Conium.
Cop. Copaiba.
Coral. Corallium rubrum.
Croc. Crocus.
Crot. Crotalus horridus.
Crot. t. Croton tiglium.
Cub. Cubeba.
Cup. Cuprum.
Cup. acet. Cuprum aceticum.
Cup. n. Cuprum nitricum.
Cup. s. Cuprum sulphuricum.
Cyc. Cyclamen.
Daph. Daphne Indica.
Der. Derris pinnata.
Diad. Aranea diadema.
Dig. Digitalis.
Dign. Digitalinum.
Dios. Dioscorea.
Dirc. Dirca palustris.
Dor. Doryphora.
Dros. Drosera.
Dulc. Dulcamara.
Elæ. Elæis guineensis.
Elaps. Elaps.
Ery. a. Eryngium aquaticum.
Ery. m. Eryngium maritimum.
Eth. Ether.
Eugen. Eugenia jambos.
Eup. per. Eupatorium perfoliatum.
Euph. Euphorbium.
Euphr. Euphrasia.
Eupi. Eupion.
Fago. Fagopyrum.
Ferr. Ferrum.
Ferr. acet. Ferrum aceticum.
Ferr. mag. } Ferrum magneticum.
Ferr. mn. }

Ferr. m. Ferrum muriaticum.
Feru. Ferula glauca.
Fran. Franzensbad.
Gad. Gadus morrhua.
Gam. Gambogia.
Gin. Ginseng.
Gran. Granatum.
Graph. Graphites.
Grat. Gratiola.
Guare. Guarea.
Gymno. Gymnocladus.
Hal. Hall.
Ham. Hamamelis.
Hell. Helleborus niger.
Hepar. Hepar sulphuris calcareum.
Hipp. Hippomanes.
Hur. Hura Brasiliensis.
Hydras. Hydrastis.
Hcy. ac. }
Hydr. ac. } Hydrocyanic acid.
Hyos. Hyoscyamus.
Hyosn. Hyoscyaminum.
Hyper. Hypericum.
Ib. Iberis.
Ign. Ignatia.
Ind. Indigo.
Inu. Inula.
Iod. Iodum.
Iodf. Iodoformum.
Ipec. Ipecacuanha.
Ir. fœ. Iris fœtidissima.
Ir. v. Iris versicolor.
Jac. Jacaranda.
Jatr. Jatropha.
Jug. c. Juglans cinerea.
Jug. r. Juglans regia.
Junc. Juncus.
Kali b. Kali bichromicum.
Kali br. Kali bromatum.
Kali c. Kali carbonicum.
Kali chlo. }
Kali clc. } Kali chloricum.
Kali iod. }
Kali hydr. } Kali iodatum.
Kali ma. Kali hypermanganicum.
Kali n. Kali nitricum. See Nitrum.
Kalm. Kalmia.
Kis. Kissengen.
Kobalt. Kobaltum.
Kreos. Kreosotum.
Lac. can. Lac caninum.
Lach. Lachesis.
Lachn. Lachnantes.
Lac. ac. Lacticum acidum.
Lact. Lactuca.
Laur. Laurocerasus.
Led. Ledum.
Lepi. Lepidium.
Lil. t. Lilium tigrinum.
Lina. Linaria.
Linu. Linum.
Lip. Lipspringe.
Lith. c. Lithium carbonicum.
Lob. Lobelia inflata.
Lob. s. Lobelia syphilitica.
Lyc. Lycopodium.
Lycper. Lycopersicum.
Lyceps. Lycopus.
Mac. Macrotinum.
Mag. c. Magnesia carbonica.
Mag. m. Magnesia muriatica.
Mag. s. Magnesia sulphurica.
Manc. Mancinella.
Mang. Manganum.
Mar. Marum verum.
Meli. Melilotus.
Ment. pi. Mentha piperita.
Meph. Mephitis.
Merc. Mercurius vivus.
Merc. c. Mercurius corrosivus.
Merc-i-fl. Mercurius iodatus flavus.
Merc-i-rub. Mercurius iodatus ruber.
Merc. s. Mercurius solubilis.
Merc. sul. Mercurius sulphuricus.
Merl. Mercurialis.
Mez. Mezereum.
Mille. Millefolium.
Mor. Morphium.
Mosch. Moschus.
Murex. Murex.
Mur. ac. Muriaticum acidum.
Myric. Myrica.
Natr. ars. Natrum arsenicicum.

Natr. bro. Natrum bromatum.
Natr. c. Natrum carbonicum.
Natr. m. Natrum muriaticum.
Natr. ph. Natrum phosphoricum.
Natr. slfc. } Natrum sulphuricum.
Natr. s. }
Nicc. Niccolum.
Nitr. ac. Nitricum acidum.
Nitr. d. s. Nitri dulcis spiritus.
Nux m. Nux moschata.
Nux v. Nux vomica.
Œna. Œnanthe.
Olean. Oleander.
Ol. an. Oleum animale.
Ol. jec. Oleum jecoris aselli.
Ol. m. See Oleum Jecoris Aselli.
Op. Opium.
Osm. Osmium.
Ox. ac. Oxalicum acidum.
Pæon. Pæonia.
Pall. Palladium.
Par. Paris quadrifolia.
Pau. p. Paullinia pinnata.
Ped. Pediculus.
Petro. Petroleum.
Phal. Phallus.
Phell. Phellandrium.
Phos. Phosphorous.
Ph. ac. Phosphoricum acidum.
Phys. Physostigma.
Phyt. Phytolacca.
Pic. ac. Picricum acidum.
Pin. s. Pinus sylvestris.
Plan. Plantago.
Plat. Platinum metallicum.
Plmbg. Plumbago littoralis.
Plumb. Plumbum metallicum.
Pod. Podophyllum.
Polyg. Polygonum.
Pru. sp. Prunus spinosa.
Psor. Psorinum.
Pte. Ptelea trifoliata.
Puls. Pulsatilla.
Puls. n. Pulsatilla nuttalliana.
Pyrus. Pyrus.
Ran. b. Ranunculus bulbosus.
Ran. sc. Ranunculus sceleratus.
Raph. Raphanus.
Rat. Ratanhia.
Rhe. Rheum.
Rei. Reinerz.
Rhod. Rhododendron.
Rhus. Rhus toxicodendron.
Rhus r. Rhus radicans.
Rhus v. Rhus venenata.
Rumex. Rumex crispus.
Ruta. Ruta.
Sabad. Sabadilla.
Sabin. Sabina.
Sac. alb. Saccharum album.
Sal. ac. Salicylicum acidum.
Samb. Sambucus.
Sang. Sanguinaria.
Sap. Saponinum.
Sars. Sarsaparilla.
Sed. Sedinha.
Selen. Selenium.
Senec. Senecio.
Seneg. Senega.
Sep. Sepia.
Sil. Silicea.
Sin. n. Sinapis nigra.
Sol. t. æ. Solanum tuberosum ægrotans.
Spig. Spigelia.
Spira. Spiranthes.
Spong. Spongia.
Squil. Squilla.
Stann. Stannum.
Staph. Staphisagria.
Stil. Stillingia sylvatica.
Stram. Stramonium.
Stront. Strontiana carbonica.
Stryc. Strychninum.
Sulph. Sulphur.
Sul. ac. Sulphuricum acidum.
Sul. iod. Sulphur iodatum.
Sum. Sumbul.
Tabac. Tabacum.
Tarax. Taraxacum.
Tarent. Tarentula.
Tax. Taxus baccata.
Tel. Tellurium.
Tep. Teplitz.

Ter. } Terebinthina.
Tereb. }
Teucr. Teucrium marum. See Marum Verum.
Thea. Thea.
Thuja. Thuja.
Ton. Tongo.
Trif. p. Trifolium pratense.
Trom. Trombidium.
Upa. Upas.
Urt. u. Urtica urens.
Ust. Ustilago.
Valer. Valeriana.
Verat. Veratrum album.
Verat. v. Veratrum viride.
Verb. Verbascum.
Vich. Vichy.
Vio. od. Viola odorata.
Vit. Vitex agnus castus.
Vös. Vöslau.
Wies. Wiesbaden.
Wild. Wildbad.
Wye. Wyethia.
Xan. Xanthoxylum.
Yuc. Yucca.
Zinc. Zincum.
Zn. s. Zincum sulphuricum.
Zing. Zingiber.
Ziz. Zizia.

